# Fabrication of multiple nanopores in a SiN_x_ membrane via controlled breakdown

**DOI:** 10.1038/s41598-018-19450-7

**Published:** 2018-01-19

**Authors:** Yunlong Wang, Cuifeng Ying, Wenyuan Zhou, Lennart de Vreede, Zhibo Liu, Jianguo Tian

**Affiliations:** 1 The Key Laboratory of Weak-Light Nonlinear Photonics, Ministry of Education, School of Physics, Nankai University, Tianjin, 300071 China; 2 Collaborative Innovation Center for Biotherapy, Nankai University, 94 Weijin Road, Tianjin, 300071 China; 3 Adolphe Merkle Institute, University of Fribourg, Chemin des Verdiers 4, CH-1700 Fribourg, Switzerland

## Abstract

This paper reports a controlled breakdown (CBD) method to fabricate multiple nanopores in a silicon nitride (SiN_x_) membrane with control over both nanopore count and nanopore diameter. Despite the stochastic process of the breakdown, we found that the nanopores created via CBD, tend to be of the same diameter. We propose a membrane resistance model to explain and control the multiple nanopores forming in the membrane. We prove that the membrane resistance can reflect the number of nanopores in the membrane and that the diameter of the nanopores is controlled by the exposure time and strength of the electric field. This controllable multiple nanopore formation via CBD avoids the utilization of complicated instruments and time-intensive manufacturing. We anticipate CBD has the potential to become a nanopore fabrication technique which, integrated into an optical setup, could be used as a high-throughput and multichannel characterization technique.

## Introduction

When we apply a voltage across a nanopore in a thin insulating membrane, the ionic current through the nanopore relates to the characteristics of particles passing through the nanopore. This way of using the Coulter Counter principle is nowadays widely used in resistive-pulse nanopore sensing^1–11^. Both biological and solid-state nanopores are used to perform DNA sensing^4,12,13^, DNA-protein interaction^14,15^, and biologically relevant molecules characterization^8,16–19^. Compared to biological nanopores, solid-state nanopores are more flexible in size and shape, easier to integrate with on-chip techniques and have a higher operating environment stability^9,20^. However, solid-state nanopores suffer from noise of the setup, limited throughput due to the resistive pulse detection principle and insufficient temporal resolution limited by the available amplifiers^4,6,21,22^. Adding optical measurement potentially overcomes the main challenges, since optical detection in a resistive-pulse nanopore sensing setup provides spectral information and spatial information, which are both independent to ionic signals with high-throughput readout^23–30^. To enable the use of optical sensing, multiple nanopores fabricated in a single membrane via a simple and cost-effective method is of great significance.

The state of the art in nanopore fabrication is either by electron beam lithography (EBL) and etching^31–33^ or by focused ion beam (FIB) drilling^34–36^. Kwok *et al*. recently reported an alternative approach to the EBL and FIB for nanopore fabrication^37–40^. By means of controlled breakdown (CBD) nanopores form in a thin membrane by applying an external potential. These nanopores can further grow to a desired diameter by monitoring the membrane conductance. So far, dielectric breakdown mainly creates single nanopores with a diameter of about 5 nm^37,40–45^. The formation of multiple nanopores requires the combination of nano- and micro- structuring^46,47^, or microfluidic channels^48^, which again increases the complexity of this method.

In this work, we present a method for multiple nanopore fabrication by CBD according to a resistance model. The nanopores formed at the same electric field strength tend to stop growing at the same diameter, based on which, we control the number and diameter of nanopores in a SiN_x_ membrane. By using fluorescence microscopy of calcium indicators, we show the potential of our multiple nanopores application in nanopore-based optical measurements. The method presented in this work significantly reduces the complexity and cost of nanopore fabrication. The method also opens a new route for solid-state nanopore fabrication without any special instrumentation.

## Results and Discussion

### The influence of breakdown electric field and time-to-breakdown

**Figure 1:**
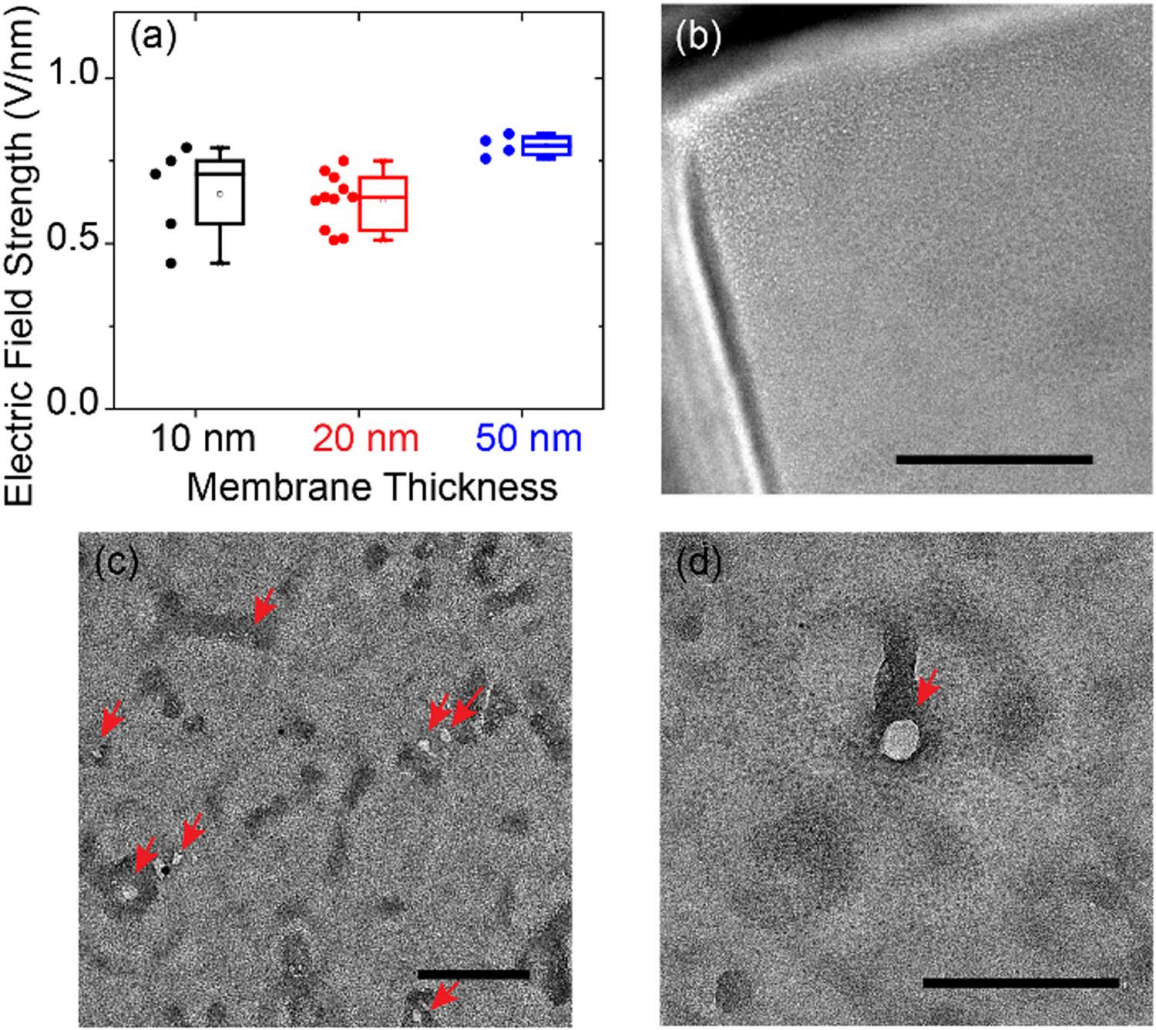
(**a**) The threshold of breakdown electric field for different SiN_x_ membrane thicknesses measured by the current-stimulus dielectric breakdown. (**b**) TEM image of a 20-nm SiN_x_ membrane without any treatment. (**c**) and (**d**), TEM images of two 20-nm SiN_x_ membranes suggest that nanopores (represented by arrows) are mostly located in the defect regions. (**b**), (**c**), and (**d**) are different membranes. Scale bar represents 100 nm.

The breakdown process depends on the material and the thickness of the membrane, as well as the time and the strength of the applied electric field^39,49–51^. In this work we control the breakdown-generated nanopore by adjusting the electric field applied across the membrane. To measure the threshold of the electric field that causes the breakdown of different membranes, we first performed the current-stimulus dielectric breakdown, as described in our previous work^44^. Figure 1(a) shows that the breakdown happened at the same range of electric field strength (0.4–0.75 V/nm) for 10 nm and 20 nm thick SiN_x_ membranes. We note that electrons (or holes) are difficult to transit through a thick membrane. To breakdown a 50 nm SiN_x_ membrane, the electric field strength should be larger than 0.75 V/nm. We cannot breakdown a 100 nm SiN_x_ membrane even when we applied an electric field of 2.0 V/nm for one hour.

**Figure 2:**
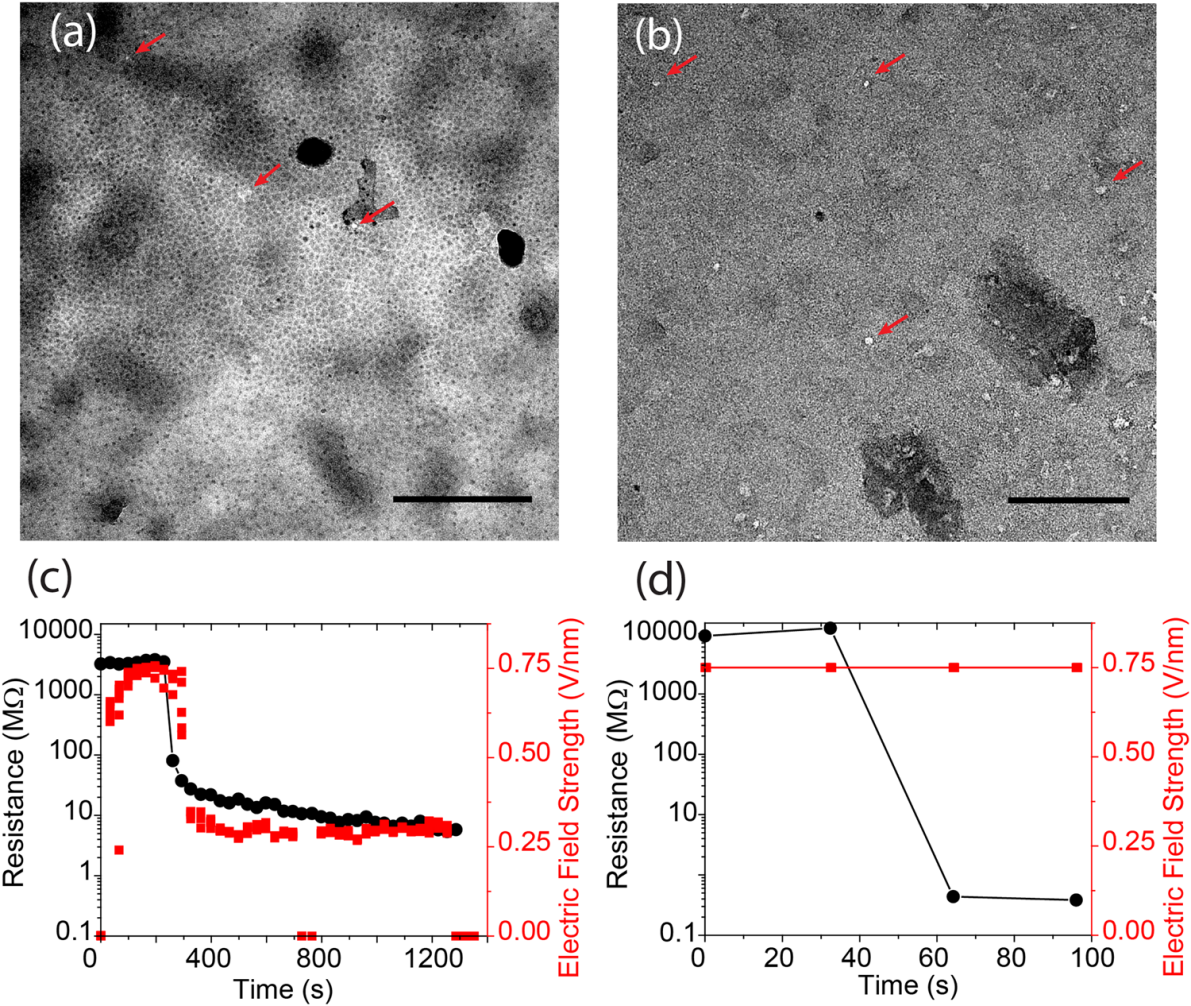
(**a**) TEM image of the SiN_x_ membrane exposed to current-stimulus dielectric breakdown in the electric field for more than 1000 seconds. (**b**) TEM image of the SiN_x_ membrane with CBD nanopores exposed in an electric field for less than 100 seconds. Scale bar represents 100 nm. (**c**) and (**d**) are the measured membrane resistance (black dots) and the strength of the applied electric field strength (red squares) during the breakdown process.

Since the conductance of the membrane does not provide any information with regard to the number or to the diameters of multiple nanopores, the electron microscopy (EM) imaging is important to gain information about nanopore distribution and the dimensions after breakdown. While scanning electron microscopy provides the detailed surface information including the nanopore shape and curvature^52^, transmission electron microscopy (TEM) has the advantage of showing whether a nanopore is through or not. This feature is important for resistive-pulse nanopore sensing, since this technique requires a through nanopore for analyte translocating through^4,10^. Figure 1(b) shows the TEM image of a 20 nm SiN_x_ membrane without any electrolyte contact. Figures 1 (c) and (d) show the SiN_x_ membranes with nanopores generated by the current-stimulus dielectric breakdown. The white spots indicate nanopores (red arrows). The dark areas are debris, which could be defects formed during high electric field stress. Figure 1 (b)–(d) suggest that the amount of defects were increasing in the membrane prior to breakdown. Nanopores are mostly located in the defect area depending on both the exposure time to the electric field as well as the strength of the electric field. To avoid the electric field slowly building up a large amount of defects in the membrane and ensure the multiple nanopore formation at the same time, we applied a high electric field over a short time period to increase the defect density and reduce the fabrication time. Figure 2 shows the exposure time influence on the 20-nm thick SiN_x_ membrane. The membrane exposed to a current-stimulus electric field for 1000 seconds shows a low contrast between nanopores and SiN_x_ membranes in Figure 2(a). While the nanopores in the membrane exposed to 0.75 V/nm for 100 seconds are distinct by the white areas in Figure 2(b). Considering the control over the membrane resistance in all the work presented here, we applied an electric field strength of 0.6 V/nm to induce a fast breakdown and decreased the electric field strength after breakdown for a controlled nanopore diameter growth. In order to create a symmetric nanochannel, we applied bipolar electric field pulses according to our previous study^44^. The use of bipolar electric field pulses is also critical for some resistive-pulse nanopore sensing applications^8,44,53^.

Previous studies of dielectric breakdown show that under the constant presence of a high electric field, the electrons flow through an insulating film when the membrane thickness is one to tens of nanometers. The electron flow causes a measurable tunneling current^41,52–54^. Before nanopore breakthrough, there are initial defects in the membrane, including the intrinsic defects randomly distributed in the ceramic material. Also electrolyte injection can lead to defects which are generated by the electric-charge difference at the membrane surfaces^54^. These defects assist the current tunneling through the membrane and accelerate the generation of defects nearby^49,50^. Some of these defects grow under the electric field stress, and eventually become nanopores when the defect density reaches the critical value^49,54^.

### Nanopore count and nanopore diameter in different electric field strengths

**Figure 3:**
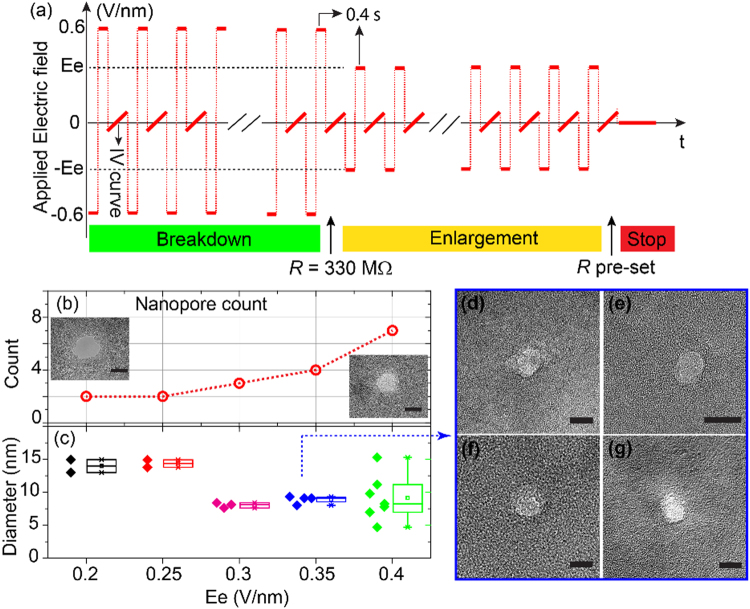
(**a**) Applied electric field during the multiple-nanopore fabrication. *Ee* represents the strength of the applied electric field during the enlargement process. (**b**) The nanopore count in the membrane at different electric fields strengths. The insets show two examples of TEM images found in two membranes enlarged at an electric field of 0.2 V/nm (left) and of 0.4 V/nm (right). Scale bar represents 10 nm. (**c**) The distribution of nanopore diameters in different membranes as a function of enlargement electric fields. (**d**–**g**) TEM images of four nanopores, enlarged at 0.35 V/nm, are found in the membrane with comparable diameters. Scale bar represents 10 nm.

Before the breakdown the initial defects are randomly distributed in the membrane and increase the defect density over time under the electric field. After the first nanopore forms, the electric field concentrates near the breakdown path due to the high conductivity of the connected electrolyte. Although the electric field strength decreased in other regions of the membrane, the existing defects keep growing in number. This growth allows the multiple nanopore formation^46,47^. To control the multiple nanopore formation process by adjusting the applied electric field, we consider the fabrication process a breakdown and an enlargement process. Figure 3(a) illustrates the applied electric field across the membrane during multiple nanopore fabrication, which is the membrane thickness divided by the applied voltage. We first applied electric field pulses of ±0.6 V/nm with a duration of 0.4 seconds for each polarity to generate nanopores. Once the resistance reached 330 MΩ at least one nanopore is formed. We reduced the electric field strength to *Ee*, which is adjusted to different values, until the resistance decreased to the pre-set value of 12 MΩ. Figures 3(b) and (c) show the nanopore count in membranes with different electric fields strengths as well as the size distributions of nanopores. As expected, a higher electric field generated defects faster and produced nanopores in the membrane in a shorter time. Meanwhile, each newly formed nanopore changes the electric field distribution in the membrane, and reduces the electric field intensity around the initial pore. This redistribution of electrical field slows down and finally stops the growth of the initial nanopore. The nanopore diameter distribution for different electric field strengths in Figure 3(c) shows that an increase of nanopores in a membrane gives smaller diameter nanopores.

Due to the redistribution of the electric field in the membrane, the nanopores formed in a membrane stop growing when the diameters of all the nanopores are equal. Figure 3(d–g) are TEM images of four nanopores formed at electric field strength of 0.35 V/nm. The diameters of these nanopores are approximately 10 nm. Depending on the electric field strength, the nanopore diameter can grow to between 10 nm and 15 nm. However, when enlarged by an electric field of 0.4 V/nm, the nanopore diameter ranges from about 5 nm to about 15 nm. We kept the applied electric field below 0.4 V/nm to ensure a narrow size distribution of the nanopore diameter enlargement.

Note that we estimated the nanopore diameters by area-equivalent diameter since the cross section shape of nanopores is not perfectly circular, for example, the left inset in Figure 3(b)^55^. For these non-circular nanopores, we measure the longer diameter (major axis) *a* and the shorter diameter (minor axis) *b* to decide the effective diameter *d* = $\sqrt{{ab}}$. This estimation is based on that the resistive-pulse nanopore sensing corresponds to the number of ions blocked in the nanopore when an analyte passes through it. We attribute the non-circular cross-section shape of the nanopores to material inhomogeneity. When the initial breakdown path is formed the electric field localizes at the breakdown path and decays with cylindrical symmetry due to electron diffusion in the SiN_x_ material^49^. In case of a defect nearby the initial breakdown location, the nanopore expands to the closest defect. This results in an non-circular nanopore shape.

### Nanopore count controlled by membrane resistance

It is unlikely that multiple nanopores form at a same time. We hypothesize that we can control the nanopore count and nanopore diameter by monitoring the membrane resistance over time. Multiple nanopores in a membrane affect the resistance in parallel, so the total resistance of membrane is,

1 $$\frac{1}{R}=\frac{1}{{R}_{1}}+\frac{1}{{R}_{2}}+\mathrm{..}.+\frac{1}{{R}_{n}},(n > 1)$$

Here *R*_*n*_ is the resistance of a single nanopore. Since we applied bipolar electric field pulses during the CBD, the diameters of the generated nanopores should mostly be symmetric^44^. The resistance of the nanopore is determined by using its effective diameter *d*_*n*_ in the conductance formula for cylindrical nanopores^56^.

2 $${R}_{n}=\frac{4t+\pi {d}_{n}}{\pi \sigma {d}_{n}^{2}}$$

Here *t* is the membrane thickness, and *σ* is the conductivity of the electrolyte. Assuming each nanopore has the same effective diameter *d*_*n*_, which is determined by the electric field strength, as shown in Figure 3(b) and (c). The number of nanopores *N* can be written as

3 $$N=\frac{R}{{R}_{n}}$$

Once we are able to control the diameters of the nanopores by adjusting the electric field strength we determine the nanopore count by the total resistance *R*.

**Figure 4:**
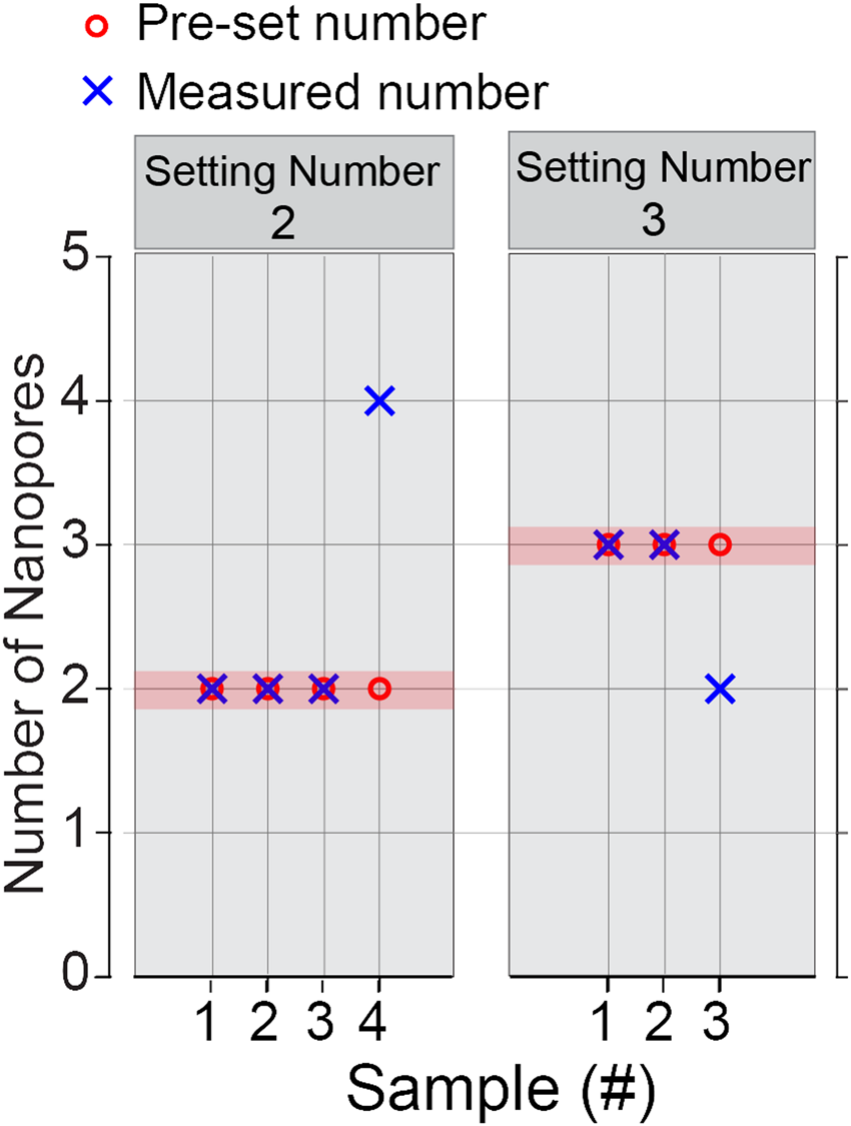
Controlling the nanopore count in the membrane according to Equation (3).

The results in Figure 3(c) indicate that nanopores enlarged at 0.3 V/nm and 0.35V/nm have the same final diameter of about 10 nm and nanopores enlarged at 0.2 V/nm and 0.25 V/ nm have the same final diameter of about 15 nm. As describe in Figure 3(a), we started our multiple nanopore fabrication by  applying an electric field of 0.6 V/nm across the membrane until the resistance of the membrane decreases to 330 MΩ, then we adjusted the electric field strength in the enlargement process and the final membrane resistance to control the number of nanopores. For fabrication of two nanopores, we applied an  electric field of 0.2 V/nm or 0.25 V/nm to increase the nanopore diameter and stopped the electric field when the resistance decreased to 9.4 MΩ. This resistance is equal to the total resistance of two 15 nm diameter nanopores. To generate three nanopores, we applied an electric field of 0.3 V/nm or 0.35 V/nm during the enlargement process and stopped when the resistance reached 12.4 MΩ, which is the total resistance of three 10 nm nanopores. Figure 4 shows that the generated nanopore count in the membrane matched our expectation.

### DNA translocations through nanopores

**Figure 5:**
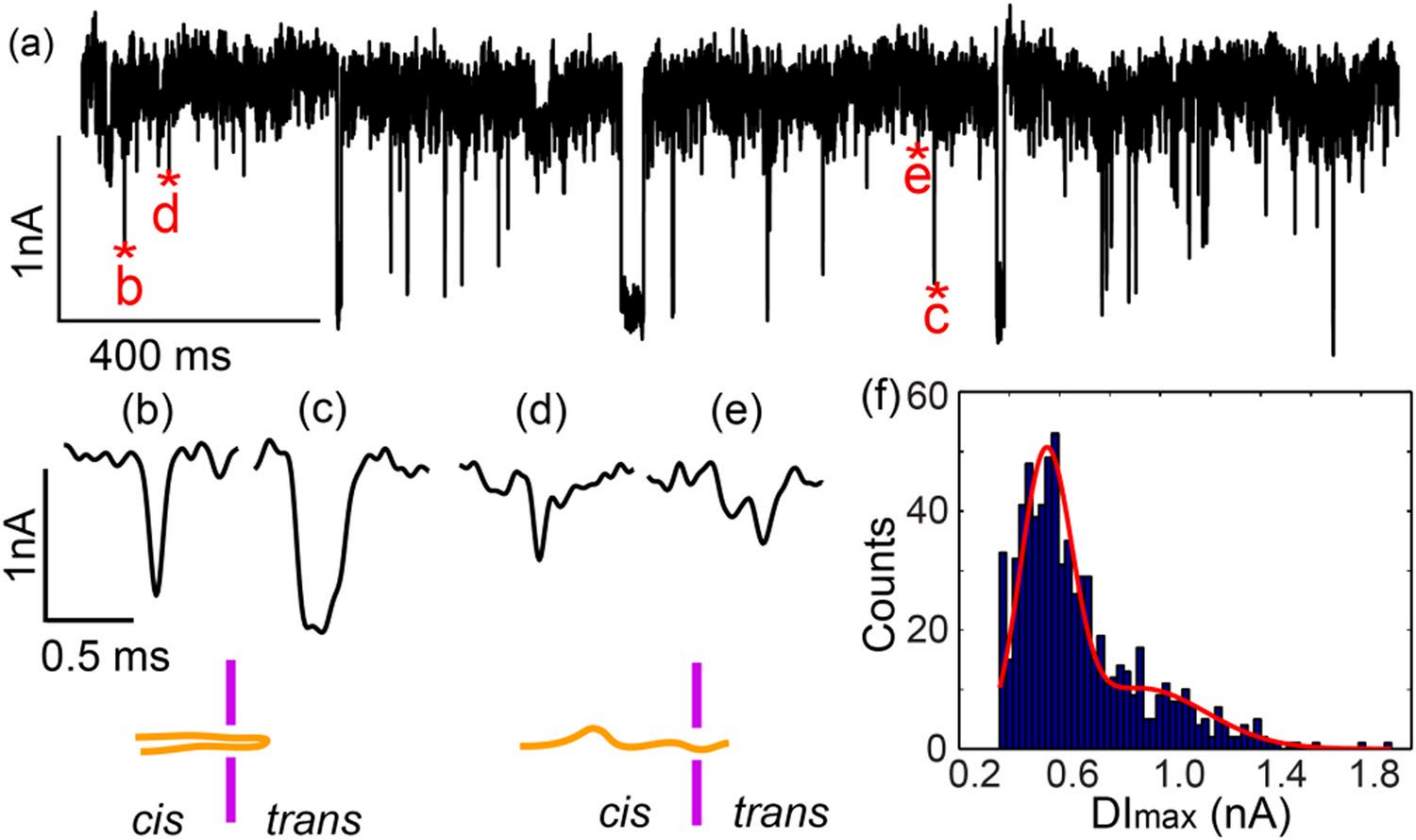
(**a**) 2-second current trace of 100 bps and 500 bps dsDNA translocation experiment. (**b**–**e**) Four typical current pulses corresponding to (**b**–**e**) in Figure 5(a). The inset illustrates different folding states of DNA molecules translocating through a nanopore. (**f**) Histogram distribution of the maximum blockade current along with two peaks Gaussian fitting. The data was collected at an applied bias of 300 mV with a signal bandwidth of 250 kHz and low-pass filter at 100 kHz.

To ensure through nanopores in the membrane we performed a double-stranded DNA (dsDNA) translocation experiment. The membrane with nanopores was loaded in a *trans-cis* flowcell and we added two kinds of dsDNA molecules (100 bps and 500 bps) to the *cis* chamber of the flow cell. The nanopore was fresh made with an estimated diameter of 5 nm based on the membrane resistance^56^. Figure 5(a) is a 2-second current trace of DNA translocation. Since there were two types of dsDNA in the *cis* chamber, this 2-second trace shows four typical events: DNA molecule translocation through nanopore in a folded structure, shown in Figure 5(b–c), and translocation through the nanopore in a chain structure, shown in Figure 5(d–e). Two peaks Gaussian fit of the histogram of blockade current^57^, *ΔΙ*, indicates these two translocation states as well, as given in Figure 5(f). This result demonstrated the capability of the CBD nanopores for nanopore sensing.

### Multiple nanopores combined with optical measurement

**Figure 6:**
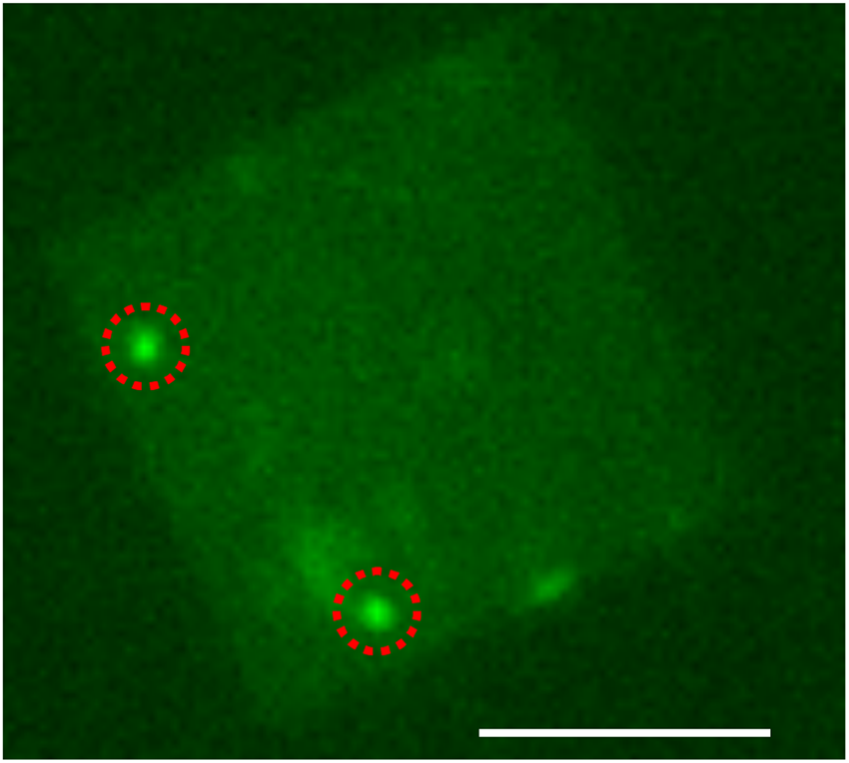
Fluorescence microscopy image of a membrane with 6.5 μM Fluo-8 in the *cis* chamber and 65 mM CaCl_2_ in the *trans* chamber at 300 mV. Two bright spots (red circles) indicate two nanopores in the membrane. Scale bar represents 10 µm.

Ca^2+^ activated dyes have recently been used for optical detection of unlabeled DNA molecules in both biological nanopores, and solid-state nanopores^23,24,46^. In this work we combine our multiple nanopores with the optical detection of Ca^2+^ activated dyes to demonstrate the potential application of our multiple nanopore fabrication in a high-throughput measurement^23,24,46^. We loaded the Ca^2+^ ions and Ca^2+^ indicator (Fluo-8) into two fluidic compartments, separated by a SiN_x_ membrane with two nanopores. When a potential is applied across the opposite membrane, Ca^2+^ ions flow through these two nanopores. These Ca^2+^ ions activate Fluo-8 when passing into the chamber. We observed two bright spots in Figure 6 by fluorescence microscopy. The brightness of these two fluorescence spots indicates that the nanopores have similar diameters.

To avoid multiple nanopores forming in a small region, control over the nanopore distribution may be necessary to meet the Abbe diffraction limit of the optical system. We have induced defects via TEM and further measured the time-to-breakdown compared to an untreated sample. A faster breakdown of the TEM-treated membrane confirmed that the nanopore can be localized in specific regions by inducing defects (Supplementary Information, Figure S1). Moreover, Zrehen *et* *al*. and Carlsen *et* *al*. reported that the breakdown can be localized in a selectively thinned membrane^46,47^. The spatial control over nanopore formation, combined with the nanopore count and diameter control pave the way for this type of nanopore array fabrication to find applications in optical measurement setups.

### Conclusions

We have shown control of the nanopore count and nanopore diameter created in a SiN_x_ membrane via controlled breakdown (CBD) by adjusting the electric field strength and the total resistance. We proposed a membrane resistance model in which we have successfully shown fabrication of multiple nanopores in a membrane with determined nanopore count and nanopore diameter. Since CBD is an accessible method for single nanopore fabrication, with precise size control, our work contributed to the CBD technology by allowing multiple nanopore fabrication. Although nanopores form in random positions during breakdown, we showed that TEM-induced defects could localize the nanopore in a specific region. By further combining surface patterning techniques, i.e. particle assembly or laser irradiation, CBD can be used to create nanopore arrays to meet the high-throughput optical measurement requirement.

## Materials and Methods

### Materials

We purchased these SiN_x_ chips as the TEM frame from Shanghai NTI Co., Ltd. (http://www.shnti.com). The SiN_x_ chips we used in this paper were 3-mm-diameter, 200-μm thick silicon frame, with 20-nm freestanding SiN_x_ membrane (by low-pressure chemical vapour deposition) located in the center (window size is around 10 μm × 10 μm). The DNA molecules were purchased from Thermo Scientific.

### Mulitple-nanopore fabrication procedures

#### Chip mounting

The electrolyte used for breakdown included 1 M KCl and 10 mM Tris-HCl (BBI Life Sciences) dissolved in the distilled water with pH 8.07. The conductivity of the electrolyte is 9.55 S/m. Before mounting the chips, we cleaned the chips in fresh made Piranha solution (by mixing H_2_SO_4_ with H_2_O_2_ with volume ratio of 3:1) for at least 30 min. We mounted a chip in a custom polymethyl methacrylate (PMMA) cell and sealed it with two silicon O-rings to separate two 200-μL fluidic reservoirs with the electrolyte solution. Two Ag/AgCl electrodes, prepared by immersion of silver wires (0.5 mm thick) into the bleacher for at least 1 hour, were placed into both reservoirs and connected to a source meter (Keithley 2450, Keithley Instruments Inc.). All setups are located in a grounded Faraday cage to isolate any electromagnetic noise.

#### Controlled breakdown

A program, written in LabVIEW (National Instruments, Austin, TX), controlled the applied voltage pulses across the SiN_x_ membrane. In this work, we divided the fabrication process to two parts, namely, breakdown and nanopore enlargement. The electric field applied across the SiN_x_ membrane is illustrated in Figure 3(a).

1. We applied high electric field pulses of ±0.6 V/nm with duration of 800 ms to create nanopores.
2. The membrane resistance was measured by Current-Voltage (IV) curve between pulses.
3. The breakdown occurred after 10–15 pulses with total breakdown time of ~250 s, as indicated by a sudden decrease of resistance.
4. Once the resistance reached to 330 MΩ, the electric field decreased to the ± *Ee* (enlargement electric field), which we adjusted according to the desired nanopore diameter.
5. As the resistance kept decreasing until reached the pre-set value, which is the *R* in Eq. (3), the program stopped applying an electric field. The whole process completed. The time of enlargement varied from different electric fields.

#### Determination of the breakdown voltage treshold

We performed current-stimulus dielectric breakdown as described in our previous work^44^. Bipolar current pulses were applied across the membrane, with magnitude of 2 μA and duration of 0.4 s for each polarity. The breakdown happed when a sudden decrease of the measured voltage was observed during the current pulses. We considered the largest voltage measured in the current-stimulus dielectric breakdown as the threshold of the breakdown voltage.

### TEM imaging

We cleaned the residual salt from the membrane by keeping the chips in distilled water for at least 2 hours after breakdown fabrication. We measured all TEM images by Tecnai G2 F20 at an accelerating voltage of 200 keV.

### Data acquisition and analysis

The buffer for DNA sensing was 1 M KCl and 10 mM Tris-HCl (BBI Life Sciences) with pH 8.07 and conductivity 9.55 S/m. We used Ag/AgCl electrodes to record ionic currents through a fresh breakdown nanopore with a patch-clamp amplifier (HEKA EPC 10, Elektronik, Harvard Bioscience, Inc.) in voltage clamp mode. All the DNA translocation data were acquired at 250 kHz, low-pass filtered at 100 kHz, and then analyzed with 15 kHz Gauss filtering using the Matlab GUI - based package - Translyser (https://github.com/voyn/transalyzer/, Calin Plesa ^57^). The minimum and maximum dwell times for search events are 0.03 ms and 3 ms, respectively. We extracted the maximum blockade currents for histograms and fitted these data by two peaks Gaussian fitting.

### Ca^2+^ - activated fluorescence microscopy

An inverted fluorescence microscope (Nikon Eclipse Ti, Nikon, Japan) measured the fluorescence microscopy image. We used the buffer according to the work of Larkin  *et al*.^24^. The electrolyte in the *cis* compartment of the fluidic cell contained 0.4 M KCl, 1 mM EDTA, 65 μM EGTA, 6.5 μM Fluo-8 (KeyGEN BioTECH, Nanjing, China) and 10 mM Tris with pH of 7.9, and the electrolyte in the *trans* component contained the 0.4 M KCl, 65 mM CaCl_2_, 10 mM Tris with pH of 7.9. During the fluorescence microscopy imaging, we applied 300 mV across the membrane. We excited the Ca^2+^ indicator by a mercury lamp and measured the fluorescence intensity with an EMCCD (ANDOR iXon3, Andor Technology), with an exposure time of 300 ms and a gain of 260.

## Electronic supplementary material

Supplementary Information
