# Supplementary material for: Fabrication of multiple nanopores in a SiNx membrane via controlled breakdown
Source: Sci Rep. 2018 Jan 19;8:1234. doi: 10.1038/s41598-018-19450-7 (PMC5775244; doi:10.1038/s41598-018-19450-7)
Supplement: Supplementary file 1 — Supplementary Information [file 41598_2018_19450_MOESM1_ESM.pdf]

Supplementary Information

Fabrication of multiple nanopores in a SiNx membrane via controlled breakdown

Yunlong Wang[1], Cuifeng Ying[1,2,3,*], Wenyuan Zhou[1,2,*], Lennart de Vreede[3], Zhibo Liu[1,2], and Jianguo Tian[1,2]

1, The Key Laboratory of Weak-Light Nonlinear Photonics, Ministry of Education, School of Physics, Nankai University, Tianjin 300071, China

2, Collaborative Innovation Center for Biotherapy, Nankai University, 94 Weijin Road, Tianjin 300071, China

3, Adolphe Merkle Institute, University of Fribourg, Chemin des Verdiers 4, CH-1700 Fribourg, Switzerland.

E-mail: cfying@nankai.edu.cn, wyzhou@nankai.edu.cn

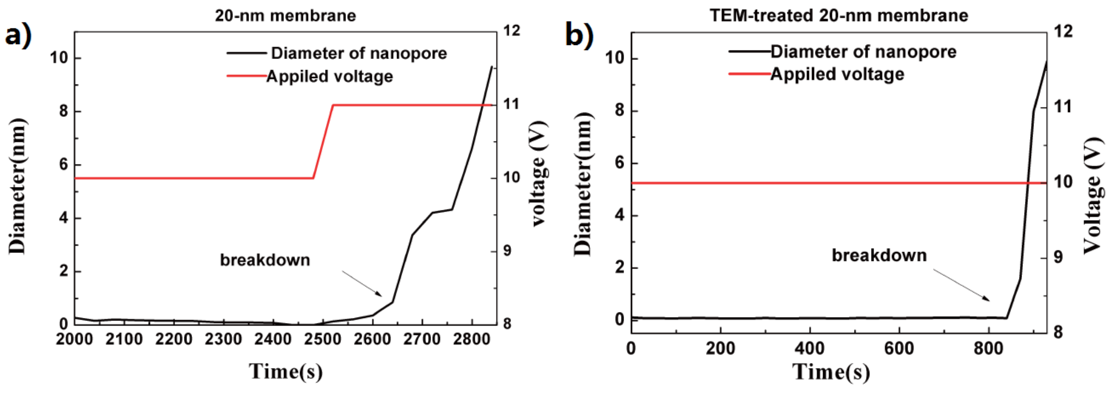

Figure S1. Time traces of voltage (red line) and derived nanopore diameter (black line) for (a) a 20-nm thick membrane and (b) TEM-treated membrane in dielectric breakdown process. For normal sample, no breakdown happens after applying 10 V for a long time (at least 2 hour). A nanopore formed after applying 11 V for 2650 seconds. While for a TEM-treated membrane, breakdown happens in 800s after applying 10 V, which is at least 4 times faster than normal sample.
